# Assessment of genetic variation for the LINE-1 retrotransposon from next generation sequence data

**DOI:** 10.1186/1471-2105-11-S9-S12

**Published:** 2010-10-28

**Authors:** Eric Rouchka, Diego E Montoya-Durango, Vilius Stribinskis, Kenneth Ramos, Ted Kalbfleisch

**Affiliations:** 1 Computer Engineering and Computer Science Department, Speed School of Engineering, University of Louisville, Louisville, KY 40292, USA; 2 Biochemistry and Molecular Biology Department, School of Medicine, University of Louisville, Louisville, KY 40292, USA

## Abstract

**Background:**

In humans, copies of the Long Interspersed Nuclear Element 1 (LINE-1) retrotransposon comprise 21% of the reference genome, and have been shown to modulate expression and produce novel splice isoforms of transcripts from genes that span or neighbor the LINE-1 insertion site.

**Results:**

In this work, newly released pilot data from the 1000 Genomes Project is analyzed to detect previously unreported full length insertions of the retrotransposon LINE-1. By direct analysis of the sequence data, we have identified 22 previously unreported LINE-1 insertion sites within the sequence data reported for a mother/father/daughter trio.

**Conclusions:**

It is demonstrated here that next generation sequencing data, as well as emerging high quality datasets from individual genome projects allow us to assess the amount of heterogeneity with respect to the LINE-1 retrotransposon amongst humans, and provide us with a wealth of testable hypotheses as to the impact that this diversity may have on the health of individuals and populations.

## Background

The Long Interspersed Nuclear Element 1 (LINE-1) retrotransposon is a repeat sequence that comprises roughly 21% of the human genome [1]. As many as 100 full length copies of the LINE-1 element have been demonstrated to be active and capable of retrotransposition within the human genome [2]. These genetic elements provide a dynamic and pervasive mechanism by which the genome may mutate, and through which it may evolve [1,3]. Several LINE-1 elements have been implicated as disease causing or mediating agents [4]. Since LINE-1 is retrotranspositionally active it is expected that analyses of emerging personal genome data would reveal a great many insertions and deletions relative to the published reference sequence for the human genome. Projects such as the 1000 genomes project provide an unprecedented opportunity to analyze the complete genomes of large populations in order to assess the amount of diversity present among them. In this work, pilot trace data from the 1000 genomes project (http://www.1000genomes.org) is analyzed for genetic diversity in terms of sequence elements that suggest full length, and therefore potentially active insertions of the LINE-1 retrotransposon relative to the published reference sequence for the human genome. Twenty-two of these elements have been identified in this work. Next generation sequencing technology is emerging as an affordable tool that will allow clinicians and researchers to develop a comprehensive picture of an individual’s genetic composition. The results of this study demonstrate clearly that as the medical field moves toward personalizing medical treatment, it will be important to assay an individual’s genome for LINE-1 elements unique to them that may prove disruptive to normal cellular function.

Retrotransposons are genetic elements that are capable of replicating themselves throughout the genome. The process of retrotransposition results in the *de-novo* insertion of either a full length, or truncated copy of the retrotransposing element into an individual’s genomic DNA. Previously unreported insertions can be found when analyzing a diverse human population [5], and have even been detected when comparing human genome assemblies from different sources [6]. In a study similar to the work presented here, it is reported that the majority of intermediate length structural variation that distinguishes inbred mouse strains from one another was caused by “…endogenous retrotransposition, predominantly by L1 retrotransposons” [7].

The full length LINE-1 retrotransposon is approximately 6000 nucleotides in length, and is characterized by two open reading frames, referred to in the literature as ORF1 and ORF2, which code for the proteins necessary to support the retrotransposition process [8]. Specific LINE-1 alleles have been identified as a disease causing or mediating agents (ref [4] and references therein). Although the function of the majority of the genome is not yet understood, it is clear that a retrotransposition event could introduce damage by inserting LINE-1 into an exon, or untranslated regions of the genome that may have regulatory function, and as a result modify a transcript, or modulate expression [9]. Also, it has been described [9-12] that LINE-1 contains the necessary functional elements, namely a 5′ UTR promoter that is capable of promoting transcription in either direction, that would drive not only the expression of LINE-1, but perhaps also of the genes that neighbor it. Although no specific evidence has been reported, this makes plausible the notion of ectopic expression of genes to which the LINE-1 insertion is proximal, or intragenic [9]. Furthermore, it has been reported that LINE-1 expression is increased dramatically under exposure to UV radiation [13], and under insult of the common environmental pollutant benzo(a)pyrene [14,15]. Increased expression of this element either endogenously, or due to environmental insult would not only serve to increase its ability to effect the expression of neighboring genes, but would also increase the likelihood of retrotransposition and, therefore possibility of somatic mutation. [3] Since this mobile element has been tied to disease, and clearly has the potential to wreak havoc with each retrotransposition event (the rate of which has estimates of between 1 in 30 and 1 in 500 in human germ cells [2]), it is clear that this is an element that must be accounted for when assessing an individual’s genetic composition.

## Results

In this study, only putative full-length LINE-1 insertion events were investigated. The reason for this is twofold. First, the full-length LINE-1 insertions are likely to have the greatest functional impact on their region of the genome. Secondly, the vast majority of the LINE-1s that are found in the genome are truncated and are missing the 5′ end. Identifying those reads that contained an insertion site for a truncated copy of LINE-1 is a more complex challenge as it is not clear that a read that contains LINE-1 sequence will also contain an insertion site, or will otherwise be comprised completely of LINE-1 sequence. By limiting the search to the 5′ end of the LINE-1 transcript, it was guaranteed that each read that contained this sequence segment would also contain the insertion site. It should be noted that LINE-1 deletion events were not considered in this phase of the project.

The search of trace data for the NA12878 sample revealed 22 insertions relative to build 36.3 of the human genome (Table 1). The trace data for the parents, NA12891 (Father) and NA12892(Mother) was searched using the insertion site flanking sequence 5′ to the LINE-1 element to determine if the insertion was inherited or if it occurred *de-novo*. No examples of *de-novo* full length insertions in the NA12878 sample were detected. Several of the identified insertions appeared to be homozygous, that is, all reads identified which contained the flanking 5′ sequence also contain the LINE-1 insert, but these findings require additional testing to substantiate them.

**Table 1:** Description of the LINE-1 insertion sites identified in this study

Chromosome	Insert Site Position	NA12878 genotype	NA12891 genotype	NA12892 genotype	LINE-1 Orientation	Gene Locus	Entrez ID	Gene orientation
Chr_2	144021434	+/-	+/-	N	-	ARHGAP15	55843	+
Chr_3	38601086	+/-	+/-	+/-	+	SCN5A	6331	-
Chr_3	82073683	+/-	Y	N	-			
Chr_3	125073416	Y	+/-	Y	+	MYLK	4638	-
Chr_3	187854832	+/-	N	Y	+			
Chr_4	132401098	+/-	+/-	+/-	-			
Chr_4	147444745	+/-	N	Y	+	SLC10A7	84068	-
Chr_5 (*)	21243489	Y	+/-	+/-	+			
Chr_5	89486537	Y	Y	Y	+			
Chr_6	102952779	+/-	+/-	N	-			
Chr_6 (*)	123895635	+/-	+/-	N	-	TRDN	10345	-
Chr_7 (*)	7985552	+/-	N	+/-	+	GLCCI1	113263	+
Chr_7	53619996	+/-	Y	?/?	-			
Chr_10	124445220	Y	+/-	Y	+			
Chr_11	109883089	Y	N	+/-	-			
Chr_12	125368882	+/-	N	+/-	-			
Chr_13	60360333	Y	Y	+/-	-			
Chr_14	51737509	Y	Y	Y	-			
Chr_15	31819131	Y	+/-	+/-	-	RYR3	6263	+
Chr_15	54038444	+/-	N	+/-	+	NEDD4	4734	-
Chr_17 (*)	62067756	+/-	+/-	N	+	PRKCA	5578	+
Chr_18	12481262	+/-	N	+/-	+	SPIRE1	56907	-

Legend: Position and genotype for the twenty two LINE-1 putative full length insertions identified in this work. The splice site position is the position of the base nearest the 5′ most base of the LINE-1 insertion in the human genome assembly build 36.3. A ‘Y’ genotype indicates that all reads found for that individual at that position contained the LINE-1 insert. An ‘N’ genotype indicates that no reads found for that individual at that position contained the LINE-1 insert. A ‘+/-‘ genotype indicates the individual was heterozygous at that position, and ‘?/?’ indicates that no reads could be found for that individual at that position. The L1 orientation of + and – indicate sense and antisense insertion respectively with respect to the assembly. Those rows marked with an (*) were verified by PCR.

**Figure 1:**
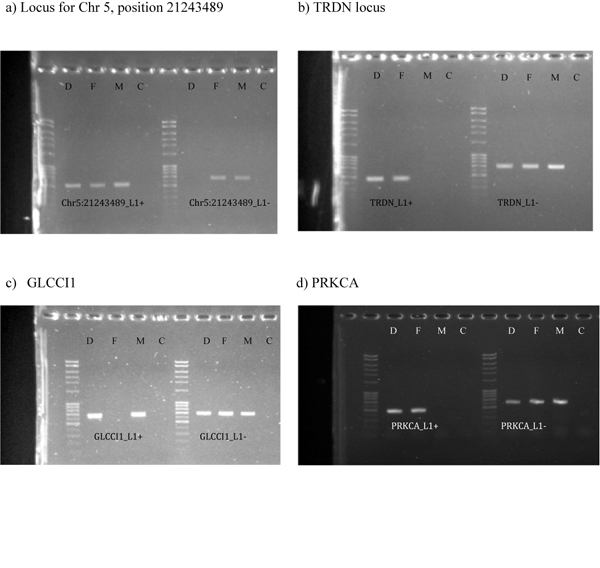
**Allele specific confirmation of genotypes**. Gel images of four validation runs using allele specific PCR. Each lane is labeled with a sample identifier D=NA12878(daughter), F=NA12891(father), M=NA12892(mother), and C=Control. For each locus, the samples were run against two pairs of primers, one that would amplify DNA that contained a LINE1 insertion (L1+), and one that would amplify DNA that did not (L1-). The four insertion sites chosen for validation were a) the intergenic region on chromosome 5 located at position 21243489 on human genome build 36.3, and those insertions within the loci for b)TRDN, c) GLCCI1, and d) PRKCA. See the Methods section for more details regarding the primer sequences and PCR conditions.

Of the 22 insertion sites identified for NA12878, 10 of the inserts are intragenic, and of those, 3 were located on the sense strand relative to the gene in which they were inserted. We chose to validate the 3 sense intragenic insertions (PRKCA, GLCCI1, and TRDN in Table 1) via allele specific PCR, as well as an intergenic insertion (position 21243489 on chromosome 5) for which there was no strong evidence within the sequence data to conclude that the insertion was inherited. LINE1+ and LINE1- alleles identified in the sequencing data were confirmed via allele specific PCR using methods described in the methods section, similar to those reported in Bennett et al.[5] The gel images are shown in Figure 1. The PCR products produced confirmed the results derived from the sequencing data, and confirmed that the insertion at position 21243489 on chromosome 5 for the daughter was inherited.

Finally, it should be noted that the decision to utilize the exact string match criteria described in the methods section will prove restrictive with respect to the comprehensive identification of all novel LINE-1 insertions in these datasets, both in terms of full length inserts that vary, even by a single base, from the search subsequence, as well as for even modestly truncated LINE-1 inserts. The manifold of subsequences that would have been necessary for a comprehensive search using this exact string match criteria would have been very large. This is a shortcoming of the search strategy that will soon be alleviated as next generation sequencing technologies improve and read lengths of 70 to 500 nucleotides become available from the 1000 Genomes project and other whole genome sequencing efforts. At that point, more comprehensive search strategies as described in the methods section that are tolerant of both sequence variation, and LINE-1 truncation will be possible since the longer reads that span LINE-1 splice sites will be more likely to contain enough LINE-1 sequence to confidently identify high fidelity matches to LINE-1, as well as sufficient flanking sequence to uniquely place the insert site in the genome. Specifically, we will be able to employ robust search and alignment tools such as BLAST[16,17] and BLAT[18] instead of the exact string match. However, even using the exact string match criteria we successfully identified 22 putative full length insertions that do not appear in the reference genome which is suggestive of hundreds of insertions that will be identifiable when using more comprehensive search strategies.

## Discussion

As the price of sequencing is driven down by next generation technologies, the reality of personal genomes for use in research, the clinic, and personal edification is destined to become a reality. In 2007 and 2008 the results of the deep sequencing of Craig Venter [19], and James Watson [20] were reported respectively. The former was produced using Sanger technology, and the latter, next generation technology. Since then, larger scale sequencing projects have begun such as the 1000 genomes project, the personal genome project (http://www.personalgenomes.org/), and additional individual projects are being undertaken [21,22]. A finding common in each of the published individual sequencing efforts is the discovery of hundreds of thousands of previously unreported polymorphisms. Conservative estimates from the data produced in the Venter and Watson studies place the number of new SNPs in excess of 600,000, and the number of new IN/DELs in excess of 22,000 [20].

It has previously been reported that there are in excess of 7000 full-length copies of LINE-1 in the reference genome sequence [23]. In this work, 22 insertions are being reported in an individual that are consistent with full-length LINE-1 elements (i.e. putative) known to be retrotranspositionally active that have not been identified previously. However, no *de-novo* insertions of putative full-length LINE-1s were detected in this sample. The insertions detected here were identified using an exact match to a 14 nucleotide sequence for reasons that are specific to the short reads available at the time of analysis (see methods). As longer reads become available, more flexible alignment searches will be possible, and the numbers of detectable insertions will, almost certainly, increase significantly.

A limitation of current next generation sequencing technology is the ability to fully characterize these insertions. Current sequence lengths are on the order of several hundred nucleotides, and the full-length LINE-1 element containing both ORF1 and ORF2 is greater than 6000 nucleotides in length. A full characterization would include the assignment of the LINE-1 to one of the many evolutionary families known to exist within humans [24]. Full-length sequencing of the entire insert is necessary not only to assign the LINE-1 to a family, but also in order to be able to assess the integrity of the LINE-1 open reading frames, and the internal promoter elements. Mutations in these regions will significantly affect the activity of the LINE-1 element, and thus, its impact on cellular function.

Several of the LINE-1 elements identified here are intragenic, may be full length, intact elements, and thus, may be of clinical significance. It is critical that as next generation data is analyzed, care is taken not to obscure the data necessary to perform analyses such as these. It is common to mask/trim known repeats prior to mapping a trace. If masked, the high quality traces selected here would not appear anomalous, and the identification of these insertions would be impossible in the mapping process. However, if the repeat region is annotated for the read (even if it is masked), the fact that it aligns to a region with no currently known repeat region would signal the presence of an insertion. Such considerations are important, as it is currently not clear how scientists will utilize this information, whether at the level of the assembly, or if the called reads will be the basis of further analysis.

The findings of this study and those published previously make clear that as more individual genomes are sequenced there will be vast increases in the number of reported polymorphisms. The technology now exists for scientists and clinicians to perform a comprehensive assay of genetic makeup of individuals. The next step is to assess the clinical relevance of these individual differences, as evidenced by diseases/disorders of altered gene expression, genomic instability or genetic toxicity. Of general interest are mutations in genes encoding for proteins involved in basal transcriptional control and responsible for reordering regions of broad local enrichment and control of chromatin structure (BLOCS). Epigenetic silencing of LINE-1 by way of DNA methylation of CpG loci within the LINE-1 promoter [25] via DNA methyltransferases as well as the involvement of E2F/Rb/HDAC complexes in the regulation of chromatin status [26] allow for the possibility of new treatment of LINE-1-mediated human diseases, once they are identified. Epigenetic drugs may help in disease treatment via modulation of the epigenetic status of BLOCS where LINE-1 sequences act as centers for epigenetic nucleation and heterochromatin formation. For example, DNA demethylating agents such as the antineoplasic 5-AzaC, and histone deacetylase inhibitors alone or in combination could restore gene expression levels of eukaryotic genes silenced as a result of LINE-1 insertion and LINE-1-mediated epigenetic silencing.

## Conclusion

LINE-1 is an active retrotransposon that has been demonstrated to affect clinical phenotype [4,27]. In this study, twenty two previously unreported insertion sites have been identified for an individual that are consistent with full length insertions of the LINE-1 retrotransposon, 10 of them intragenic. As we move toward the paradigm of personalized medicine, it is clear that more work is necessary to fully catalogue the diversity with respect to this element within the human population. This will allow us to assess the allele frequency of all existing LINE-1s, and begin to classify them with respect to clinical relevance. This understanding will put us in a much better position to evaluate the impact of de novo insertions as they are discovered since this is an element that should be accounted for when performing genetic tests for disease causing mutations.

## Methods

### Sequence search

Pilot sequence data was downloaded from the 1000 genomes ftp site located at NCBI [28]. The genome used for all alignments and comparisons was the reference assembly from NCBI Build 36.3, specifically, those sequence files with the “hs_ref_” prefix in the directory /genomes/H_sapiens/ARCHIVE/BUILD.36.3/Assembled_chromosomes of the NCBI ftp site. The samples searched were a HapMap family trio daughter (NA12878), father (NA12891), and mother (NA12892) belonging to the CEPH/Utah pedigree with the family id of 1463 in the Coriell repository. Previously unreported LINE-1 insertion sites were detected via an exact string search within each NA12878 trace for the pattern GGGGAGGAGCCAAG and its reverse complement taken from the Human LINE-1 (L1.4) entry in GenBank (Version L19092.1; GI:307102 nucleotide positions 2-15). This sequence element is conserved in the 5′ end of the 5′ UTR of ORF1 in many of the sequences in the active L1Ta-1 family (accessions taken from reference [24]). The short sequence was chosen because much of the pilot data accessible at the time of this study was produced on platforms that produced shorter reads (48 nucleotides or shorter). In order to locate the insertion site within the genome, it was necessary to have sufficient high quality non-LINE-1 sequence within the read that flanked the insert (~25 nucleotides in length). As such, the 14 nucleotide search sequence often resulted in hits with enough flanking sequence, even in the shorter reads, to map the insertion site to the genome and generate additional flanking sequence from the genome consensus to search for other sequences that corroborated the insertion, or provided evidence that the insertion was heterozygous.

**Figure 2:**
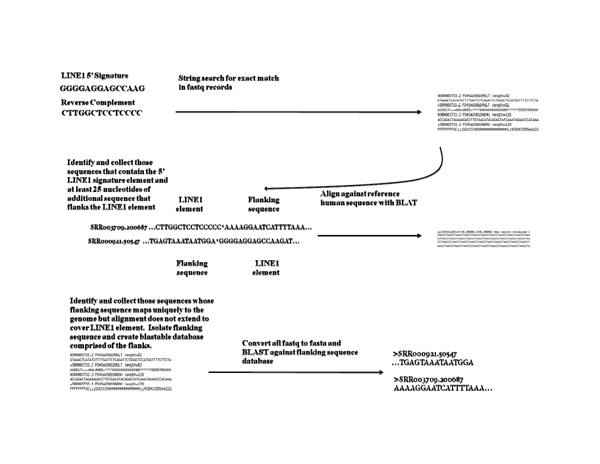
**Diagram of insert search process.** A diagram describing the sequence search process by which putative full length LINE-1 insertion sites were identified.

Those reads from the NA12878 individual that contained a putative LINE-1 insertion that did not appear in the reference assembly build 36.3 were stored. This search was performed with BLAT [18]. A total of 22 such locations were found for this sample. Regions of at least twenty-five bases in length flanking the 5′ end of these LINE-1 insertions were stored and used to search the sequence data for other traces in all samples spanning the insertion site. If the flanking region was either shorter than 25bp or was not unique in the genome, it was discarded. A blastable database containing the filtered set of 22 sequences was created using formatdb. Fastq files for the individual genome projects NA12878, NA12891, and NA12892 were distributed using the Torq scheduler’s qsub command. Each of the sequences were converted to fasta format on the fly, piped through, and compared to the blastable database using NCBI’s blastn v2.2.19 with the parameters –m 8 –W 22 to find only regions highly similar to the flanking regions. Those regions returning blast hits according to these parameters were further mapped to each of the 24 chromosomes of the human genome individually using BLAT [18]. The BLAT results were sorted according to their chromosomal location. Hits overlapping the same region were grouped together to produce a sequence alignment. The alignments for all loci can be found in the additional file 1. The location of the 5′ end of the LINE-1 element was noted by the presence of either GGGGAGG or its reverse complement CCTCCCC and marked by a ‘*’. These alignments were further visually inspected to test for the presence of homozygosity or heterozygosity of the LINE-1 element within each of the datasets for the three individuals. The process is illustrated in Figure 2. It must be noted that the dataset at the time of analysis did not provide deep coverage for all insertion sites. As such, these inspections can only offer insights with respect to hetero- or homozygosity that require further testing. In some cases in the analysis of the parent data, we were not able to identify traces with flanking data that had the 5′ LINE-1 signature. In many of those reads, a poly-A track was found that was not consistent with the corresponding locus on the genome assembly. This poly-A track was taken to be sufficient evidence of the 3′ poly-A tail of LINE-1, and the sample would have been designated as having a LINE-1 genotype. This poly-A tail condition was used only for the parents. In all cases for the daughter, the 5′ end of LINE-1 was identified in at least one read.

In a few cases, early indications suggested that insertions found in the NA12878 (daughter) were not inherited from either parent and appeared to be de novo. For these insertions, we performed ad hoc string searches that included 10 to 15 bases of unique flanking sequence as well as the first 7 bases sequence from the 5′ end of the LINE-1 insert. As such, several sequences can be found in the alignments in additional file 1 that do not pass the 25 bases of flanking sequence requirement described above.

### Justification for choice of sequence search method

The aim of this work is the identification of full-length LINE-1 insertions that do not appear in the human reference genome. As such, it was necessary to identify sequence reads amongst the pilot data that contained the LINE-1 insertion site. We define reads that contain the insertion site as those that include the 5′ end of the LINE-1 insert, as well as enough sequence flanking the insertion site that it is possible to place the insertion site uniquely in the genome. For this, we needed 25 nucleotides or more of unique, non-LINE-1 sequence within the read. The best choice for a sequence search strategy in cases such as this is one that employs an alignment search tool such as BLAST [16,17] or BLAT [18] that will identify high scoring pairs (HSPs) of alignments between subsequences of the full length LINE-1 and those reads that contain LINE-1 sequence. With sufficiently long reads (~70 nucleotides or longer), this strategy would make it possible to confidently identify those reads that contain high fidelity matches with any LINE-1 subsequence. Additionally, it would be tolerant of gaps and mismatches, and would make it possible to identify evidence of truncated LINE-1 insertions. However due to the shorter sequences, in many instances 48 or fewer nucleotides, the HSPs that would have been returned would have been difficult to interpret. Those that contained shorter LINE-1 alignments (20 or fewer nucleotides), and hence more unique non-LINE-1 sequence, were difficult to evaluate in terms of actual LINE-1 composition. For those that contained longer LINE-1 sequences it was easier to assess their LINE-1 composition, but difficult or impossible to place in the genome by virtue of the fact that they had less unique non-LINE-1 sequence. Also, polymorphisms, either single nucleotide, or insertion/deletion, combined with low quality sequence may truncate the HSP, falsely suggesting a truncated LINE-1insertion.

As described in the results section, the search for putative full length insertions is advantageous for two reasons. First, since the subsequence is the 5′ most end of the LINE-1 transcript, it is guaranteed that any read containing the subsequence will also contain the insert site. Other subsequences taken from the LINE-1 transcript presented a more difficult analysis scenario. Secondly the full length insertions are likely to have the greatest regulatory impact. The choice of a short subsequence was made to increase the likelihood that we would have sufficient non-LINE-1 sequence in any read identified to uniquely place the sequence in the genome. These issues will be mitigated when longer sequence data becomes available, and a more comprehensive search will be possible that identifies both truncated, and polymorphic insertions with confidence.

### PCR analysis

PCR was performed to confirm the presence of LINE1+ and LINE1- in four loci, chromosome 5, position 21243489, TRDN (entrez_id=10345), GLCCI1 (entrez_id= 113263), and PRKCA (entrez id = 5578). Primer3 [29] was used to design the primers for these reactions that flanked each of the four insertion sites. The sequence for L19092.1 was used as the template for the LINE-1 insertion, and a 3′ oligo was designed TGAACCCGGTACCTCAGATG, that when used with the 5′ oligo would produce a PCR product if the LINE-1 element was present in the locus as suggested by the sequence data. The full list of primer pairs used is shown in Table 2. Standard PCR conditions were used with an annealing temperature of 61.3°C for 35 amplification cycles. The Fisher Bioreagents exACTene DNA Ladders mid range plus DNA ladder was used to assess the length of the PCR products. Samples for the three individuals were obtained from the Coriell Institute.

**Table 2:** Primers used for allele specific PCR

Locus/Allele	5' Oligo	3' Oligo	PCR Product Size(nt)
Chr5:21243489_L1+	ttgcctaagcttcccataca	TGAACCCGGTACCTCAGATG	539
Chr5:21243489_L1-	ttgcctaagcttcccataca	tgatccatgtcaataatgaggtc	677
TRDN_L1+	tttcatgccatctctatattccaa	TGAACCCGGTACCTCAGATG	477
TRDN_L1-	tttcatgccatctctatattccaa	aaaaacaggcctgagattgc	690
GLCCI1_L1+	tcctcatacaaacttgtgaagtgat	TGAACCCGGTACCTCAGATG	666
GLCCI1_L1-	tcctcatacaaacttgtgaagtgat	ttcaagcttcatctaatgaaagaaaa	682
PRKCA_L1+	tgtgcaaatttcaccccata	TGAACCCGGTACCTCAGATG	527
PRKCA_L1-	tgtgcaaatttcaccccata	ccatagagcagtgacccaca	622

Legend: Primers used for the allele specific PCR verification of the next generation sequence data analysis. The same right primer was used in all L1+ PCR reactions. This primer spans bases 90 to 109 of the LINE-1 transcript with accession L19092.

## Addendum

A paper recently published by Ewing and Kazazian [30] subsequent to the original presentation of this work introduced next generation sequencing for the identification of LINE-1 inserts within individual genomes. Their analysis included the same three Caucasian samples analyzed in this work along with samples from 22 other individuals from ethnic backgrounds that included, Caucasian, Yoruban and Japanese. Of the 22 putative full length inserts identified here, 14 of them were confirmed by Ewing and Kazazian. The eight additional inserts identified here (Table 3) emphasize the need for complementary approaches to the identification of comprehensive LINE-1 insertion profiles in humans.

**Table 3:** Insertions identified by sequence search of whole genome shotgun sequence data

Chromosome	Insert Site Position	Gene Locus
Chr_2	144021434	ARHGAP15
Chr_5	21243489	
Chr_6	102952779	
Chr_7	7985552	GLCCI1
Chr_14	51737509	
Chr_15	31819131	RYR3
Chr_17	62067756	PRKCA
Chr_18	12481262	SPIRE1

Legend: The splice site position is the position of the base nearest the 5′ most base of the LINE-1 insertion in the human genome assembly build 36.3.

## List of abbreviations

LINE-1: Long interspersed nuclear element-1

## Competing interests

TK is President of Intrepid Bioinformatics, 201 East Jefferson Street, Louisville, KY 40202.

## Supplementary Material

Additional file 1 Additional file 1 Click here for file
